# Enhancing Mental Health and Psychosocial Support for Adolescents and Young Adults Through the Asenze Impilo Project: Protocol for a Human-Centered Design Study

**DOI:** 10.2196/90096

**Published:** 2026-09-10

**Authors:** Thokozile J Mbaya, Phiwe Babalo Nota, Nonjabulo Purity Gwala, Christopher M Ferraris, Christopher Magana, Kathryn Watt, G Nduku Wambua, Leslie L Davidson, Chris Desmond, Jeremy Kane, Justin Knox, Gavin George

**Affiliations:** 1 Department of Practical Theology and Missiology, Faculty of Theology, Stellenbosch University, 171 Dorp Street, Stellenbosch, Western Cape, 7600, South Africa, 27 658333112; 2 Children's Institute, Faculty of Health Sciences, University of Cape Town, Cape Town, Western Cape, South Africa; 3 Health Economics and HIV and AIDS Research Division, University of KwaZulu-Natal, Durban, KwaZulu-Natal, South Africa; 4 New York State Psychiatric Institute, New York, NY, United States; 5 Centre for Research in Health Systems, University of KwaZulu-Natal, Durban, KwaZulu-Natal, South Africa; 6 Africa Health Research Institute, Durban, KwaZulu-Natal, South Africa; 7 Department of Psychiatry, University of KwaZulu-Natal, Durban, KwaZulu-Natal, South Africa; 8 Department of Epidemiology and Pediatrics, Columbia University Irving Medical Center, New York, NY, United States; 9 Department of Epidemiology, Columbia University Mailman School of Public Health, New York, NY, United States; 10 Department of Psychiatry, Columbia University Irving Medical Centre, New York, NY, United States; 11 Department of Sociomedical Sciences, Columbia University Mailman School of Public Health, New York, NY, United States; 12 Division of Social Medicine and Global Health, Lund University, Lund, Skåne, Sweden

**Keywords:** mental health, adolescent, young adults, psychosocial support, human-centered design, co-design, implementation science, low- and middle-income countries, South Africa, qualitative research

## Abstract

**Background:**

Despite increasing recognition of adolescents’ and young adults’ mental health needs, many young people continue to experience barriers in accessing timely, acceptable, and responsive mental health and psychosocial support (PSS) services. There is a need for contextually relevant and youth-responsive approaches that meaningfully engage young people, caregivers, and service providers in implementation strategy development. The *Asenze Impilo* study aims to co-design a mental health and PSS implementation strategy for adolescents and young adults in KwaZulu-Natal (KZN), South Africa, using a human-centered design (HCD) approach.

**Objective:**

This protocol paper describes the methods used to co-design an implementation strategy to enhance existing mental health and PSS services for adolescents and young adults, leveraging resources from the Asenze study, a population-based longitudinal cohort study in KZN, South Africa. The study will use an HCD approach to engage adolescents and young adults, caregivers, and various mental health stakeholders from the Departments of Health, Basic Education, and Social Development, and implementing partner organizations in order to inform how to improve mental health for adolescents and young adults by (1) identifying needs and barriers, (2) developing context-relevant solutions, and (3) refining a co-designed implementation strategy.

**Methods:**

A 3-phase HCD co-design process will be undertaken, using qualitative multimethods. Participants will include 20 young adults recruited from the Asenze study, 20 caregivers recruited from the Asenze study, and 15 mental health stakeholders from KZN, South Africa. Phase 1, *Discover*, includes separate HCD workshops with adolescents and young adults, caregivers, and implementation partners to understand existing mental health and PSS services, mental health and PSS needs of adolescents and young adults, and potential evidence-based intervention options and corresponding implementation strategies. Phase 2, *Design*, includes separate HCD workshops with adolescents and young adults and caregivers to co-design a context-specific mental health and PSS implementation strategy. Phase 3, *Build*, includes 2 separate HCD workshops with adolescents and young adults and caregivers to refine the co-designed implementation strategy, and a consultation workshop with the mental health stakeholders to review the feasibility and acceptability of the proposed implementation strategy. A qualitative thematic analysis approach will be used, allowing for an inductive and iterative exploration of patterns emerging from the data collected during the HCD workshops.

**Results:**

The study was funded in 2024, and ethics approval was received in March 2025. Enrollment will be completed in April 2025, and data collection will be conducted between May 2025 and January 2026. Findings are expected to be submitted for publication later in 2026.

**Conclusions:**

The study aims to produce a co-designed implementation strategy to improve delivery of mental health and PSS for adolescents and young adults that is adapted to the local context.

## Introduction

### Overview

Mental health disorders are a significant global health challenge, with a disproportionate burden in low- and middle-income countries (LMICs), particularly sub-Saharan Africa (SSA), where resource limitations and unmet needs are profound [1]. These challenges have intensified in recent years, with the COVID-19 pandemic disrupting already fragile mental health systems and exacerbating psychosocial stressors, particularly among adolescents and young adults [1].

Young people in SSA face a convergence of structural and environmental risks, including poverty, exposure to violence and armed conflict, the enduring impact of the HIV/AIDS pandemic, and the disruption of education due to the COVID-19 pandemic restrictions, that heighten their vulnerability to a range of mental health conditions [2-4]. Systematic reviews have documented high prevalence rates of emotional, behavioral, and psychological difficulties among children and adolescents in the region [5-7]. These challenges are often exacerbated by experiences such as bullying, gender-based violence, substance misuse, and academic pressures.

Adolescents and young adults constitute a critical period for mental health development, necessitating timely access to effective mental health and psychosocial support (PSS) services [8]. Despite this well-documented need, access to professional mental health services in LMICs remains severely constrained, with many countries having fewer than one specialist per 100,000 population [9]. Persistent barriers such as entrenched stigma, reliance on traditional healing practices, mistrust of school-based counseling, and chronic shortages in mental health resources further exacerbate these challenges [10-12].

These persistent gaps underscore the urgency for innovative, scalable, and culturally attuned interventions. Task-shifting strategies, whereby nonspecialists such as teachers, community health workers, and peer counselors are trained to deliver evidence-based psychosocial interventions, have shown promise in mitigating workforce shortages [12,13]. Embedding such interventions within schools and community structures offers potential to reduce treatment gaps; however, there remains a paucity of systematic evidence comparing their effectiveness, implementation strategies, and adaptability across diverse SSA contexts [14]. Schools provide an important platform for delivering health interventions to adolescents and young adults broadly, and mental health services and PSS are key components of this provision [15,16]. Generating robust evidence on which interventions are most effective, for whom, and under what conditions is critical for informing policy decisions and optimizing resource allocation in resource-limited settings.

In South Africa, the unmet need for mental health services among adolescents and young adults is particularly acute [17,18]. Results of the South African National Youth Risk Behavior Survey found that 24% of respondents between grades 8 and 11 had experienced feelings of depression, hopelessness, and sadness, and 21% had attempted suicide at least once [19]. Another study, among school learners aged 14-15 years in Cape Town, reported high prevalences of depression (41%), anxiety (16%), and posttraumatic stress disorder (21%) [20]. Policies and programs in South Africa, including the 2012 Integrated School Health Policy (ISHP) and the 2014 Screening, Identification, Assessment, and Support (SIAS) Policy, have been developed with the express aim of improving both health and education outcomes among children and youth in South African schools. These policies target children, adolescents, and young adults from grades 1 to 12, between the ages of 6 and 18 years.

The ISHP is a multisectoral policy that mandates the Department of Health (DoH), Department of Education (DoE), and the Department of Social Development (DSD) to include comprehensive health screening and mental health screening using the Strengths and Difficulties Questionnaire [21]. The mental health screening is targeted at learners in grades 1, 4, 8, and 10, and this is done once in each of these grades and repeated for any learner who repeats any of these grades. The ISHP further provides for on-site services and health education in the foundation phase (grades R-3), intermediate phase (grades 4‐6), senior phase (grades 7‐9), and further education and training phase (grades 10‐12) [21]. However, gaps and inconsistencies in implementation persist, driven by shortages of trained personnel, a lack of cross-sectoral collaboration between education and health ministries, and weak referral pathways [22,23].

The SIAS Policy aims to provide additional support to learners who have been identified in mainstream and special schools with special needs and difficulties in learning, such as poverty, language, family issues, disability, and learning difficulties [24]. The policy also outlines structural arrangements to support learner inclusion, including support staff within the education district, articulating the roles of the district-based support teams, the school-based support teams, and school management. Despite the presence of these policies, the provision of mental health and PSS services remains inconsistent and suboptimal [25], requiring urgent strengthening if the mental health needs of adolescents and young adults are to be effectively addressed.

To address these persistent service delivery gaps, innovative and participatory approaches are required. Human-centered design (HCD) offers a promising framework for codeveloping mental health implementation strategies that are contextually grounded and responsive to end user needs. HCD emphasizes collaboration with individuals who have lived experience of a problem, in this case, adolescents and young adults, as well as with caregivers and service providers, to ensure that resulting implementation strategies are both feasible and culturally resonant [26,27]. As a problem-solving approach, HCD is particularly suited to complex health system challenges like those found in perirural South Africa, where user engagement is critical for the uptake, acceptability, and sustainability of mental health implementation strategies. Given the convergence of poverty, high HIV prevalence, and structural inequality in KwaZulu-Natal (KZN), HCD is deployed here not only as a design method but also as a means of empirical inquiry into the contextual, systemic, and experiential factors shaping adolescents’ and young adults’ mental health needs.

Operationalizing this HCD approach, the Asenze Impilo project is nested within the Asenze longitudinal cohort study, now in its fifth wave, which has generated robust data on adolescent development, health, and mental health in KZN over a period of more than a decade [28-31]. Prior waves of Asenze illustrate the prevalence of the common mental health disorders (CMHDs), including depression, among adolescents in the cohort, with 36% of the wave 4 sample presenting with a CMHD and 4.9% screening positive for 2 or more CMHDs [31].

At the time of their wave 4 assessment, 79% of Asenze adolescents were in school, 13% had completed matric (the National Grade 12 certificate; equivalent of completing high school), 5% had dropped out of school, and 3% were in technical and vocational education and training colleges. Despite Asenze participants being over the age of 18 years in wave 5 (the current wave of the study), the education system is likely to remain a key factor in Asenze young people’s lives because some of these young adults continue their schooling beyond 18 years. Combined with the prevalence of mental health challenges in this population and the demonstrated influence of a sense of school belonging as protective of mental health, the education system presents an important opportunity to intervene to protect mental health [32].

The Asenze Impilo study will be an application of the observational-implementation hybrid approach. Observational-implementation hybrid studies integrate implementation science methods within observational research to generate evidence that informs the development, delivery, and evaluation of implementation strategies. This approach has the potential to increase public health impact by broadening traditional observational epidemiologic study designs to include the collection of data to inform the design, implementation, or evaluation of implementation strategies on implementation outcomes, including service delivery/health outcomes. The Asenze Impilo will achieve this by incorporating implementation science methods into the observational Asenze cohort study and will collect information that will inform implementation strategies to address CMHDs among adolescents and young adults [33]. The longitudinal Asenze cohort offers a unique opportunity to conduct this work, providing over a decade of rich, contextual data that allows for an in-depth understanding of how social determinants of health, including the impact of COVID-19, affect mental health among adolescents and young adults. The Asenze cohort also provides a ready and diverse group of young adults who have lived experiences with mental health challenges and caregivers who have been previously screened that can be recruited for HCD workshops. These participants can offer critical insights into barriers and opportunities in the current system to use findings from prior research.

The Asenze Impilo study will use an HCD approach to codevelop solutions with young adults and caregivers, aligning implementation strategies with real-world challenges and contextual needs in KZN. By bringing together insights from the Asenze study, prior ISHP evaluations, to which several members of the Asenze Impilo research team contributed [25], and community members, Asenze Impilo will integrate evidence and stakeholder perspectives to co-design a tailored, context-specific, and effective implementation strategy to help address a critical implementation gap.

### Study Aims

The Asenze Impilo study aims to identify strategies for improving implementation of evidence-based mental health and PSS interventions for adolescents and young adults. This knowledge can be used to address implementation gaps and, in turn, improve mental health outcomes among adolescents and young adults in South Africa. This will be achieved by bringing together young adults from the Asenze cohort, their caregivers, stakeholders from the provincial district DoE, DoH, DSD, other implementation partners, and nongovernment organizations (NGOs) staff, to co-design a context-specific, adolescent and young adult–friendly, and culturally sensitive implementation strategy for evidence-based mental health and PSS interventions for adolescents and young adults in KZN and completing the following steps:

- Map existing mental health and PSS services for adolescents and young adults in KZN.
- Identify service delivery barriers from stakeholder perspectives.
- Co-design a context-specific implementation strategy for existing evidence-based interventions (identified during a previous systematic review) to address CMHD among adolescents and young adults with stakeholders.
- Assess feasibility and acceptability of the proposed implementation strategy.

The implementation strategy that is codeveloped can then be evaluated for acceptability, feasibility, effectiveness, and cost-effectiveness.

## Methods

### Theoretical Framework

This study is theoretically informed by the social ecological model (SEM) and operationalized through a HCD methodology [26,34,35]. The SEM provides a multilevel framework for understanding the determinants of mental health, while HCD offers a user-driven methodology for generating practical, context-specific implementation strategies. The SEM frames mental health as shaped by interrelated factors operating at 4 levels—individual, interpersonal, community, and policy—requiring multilevel strategies. At the individual level, factors such as personal experiences, mental health conditions, and coping strategies shape well-being [35]. The interpersonal level considers the influence of relationships with family, peers, and caregivers, which can either support or hinder mental health. At the community level, access to resources, social norms, stigma, and structural barriers impact the effectiveness of mental health implementation strategies. Lastly, the policy level encompasses broader systems such as government policies, health care structures, and legal frameworks that shape service availability and implementation.

The HCD approach complements this by engaging directly with individuals and stakeholders within each of these SEM levels to codevelop tailored solutions. HCD ensures that implementation strategies are culturally relevant, contextually appropriate, feasible, and sustainable for real-world application [27]. This is particularly crucial in mental health and PSS service provision, where solutions must align with local beliefs, values, and service delivery constraints to be effective. This integration ensures that implementation strategies are not only user-driven but also system-sensitive.

Each phase of the HCD process aligns with specific SEM levels (Table 1).

**Figure 1.**
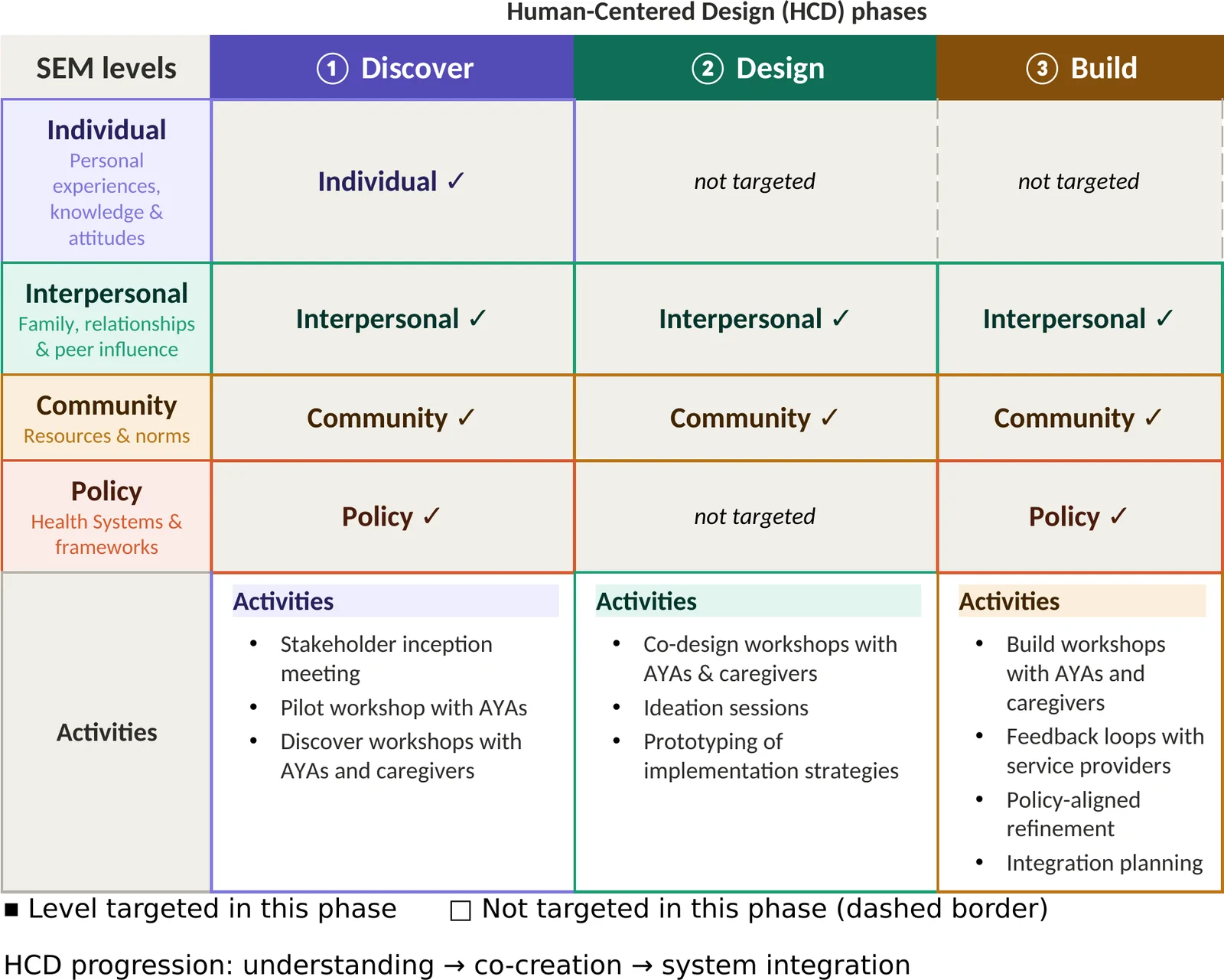
Conceptual framework illustrating the integration of the social ecological model (SEM) and human-centered design (HCD). AYA: adolescents and young adults.

This structured alignment ensures that each phase of the implementation strategy development is responsive to both user needs and the broader system context. (Figure 1).

By integrating SEM and HCD, this study adopts a holistic and participatory approach to co-designing a context-specific mental health implementation strategy. Engaging diverse stakeholders, including adolescents and young adults, caregivers, government, and their implementing partners, is essential to navigating the multilayered challenges in mental health service delivery and fostering solutions that are both effective and intended to support future scale-up within the specific sociocultural and policy environment of KZN.

**Table 1.** Alignment of the human-centered design (HCD) phases and social ecological model (SEM) targeted levels.

HCD phase	SEM targeted levels	Activities
Discover	Individual, interpersonal, community	Inception meeting with mental health stakeholders; pilot workshop with adolescents and young adults; discover workshops with adolescents and young adults and caregivers; these will include mapping lived experiences, service journey mapping, and identifying gaps
Design	Interpersonal, community	Co-design workshops with adolescents and young adults and caregivers; ideation and prototyping of implementation strategies
Build	Community, policy	Build workshops with adolescents and young adults and caregivers; feedback loops with mental health stakeholders; policy-aligned refinement and integration planning

### Study Design

#### Overview

Asenze Impilo uses an observational-implementation hybrid approach. Specifically, implementation science activities are embedded within the observational Asenze cohort study. Asenze Impilo uses a 3-phase qualitative participatory approach that integrates qualitative participatory methods to co-design a mental health and PSS implementation strategy for adolescents and young adults. The study uses HCD, a problem-solving methodology that emphasizes collaboration with end users to develop implementation strategies that are contextually relevant and sustainable [36]. By actively engaging adolescents and young adults, caregivers, and mental health service providers, this study seeks to create a locally adapted and practical implementation strategy for improving mental health care within existing health and education systems.

The first phase (Discover) focuses on understanding the current landscape of mental health services, identifying existing gaps, and exploring the experiences of key stakeholders. The second phase (Design) involves the collaborative development of an implementation strategy through a co-design workshop. The third phase (Build) entails refining and remodeling the developed strategy, assessing its feasibility, acceptability, and effectiveness. Table 2 (HCD 3-phase summary) provides a summary, and each phase is described in further detail below.

**Table 2.** Human-centered design 3-phase summary.

Phase	Objective	Activities and participants	Expected outcomes
Phase 1 (Discover)	To map existing mental health and PSS^a^ services for AYAs^b^ in KZN^c^ To identify service delivery barriers from stakeholder perspectives	Systematic review of existing services Inception meeting with 15 stakeholders (DoE^d^, DoH^e^, DSD,^f^ and NGOs)^g^ Qualitative inquiry with 20 young adults and 20 caregivers (journey mapping, FGDs)^h^ Pilot workshop with young adults to test tools	Experiences Validated data collection tools
Phase 2 (Design)	To co-design a context-specific implementation strategy with stakeholders	Present phase 1 findings to 3 groups of stakeholders Conduct separate ideation workshops with young adults and caregivers Brainstorm ideas and solutions Select most appropriate solutions and ideas through plenary session	Consensus on preferred and best ideas for implementation
Phase 3 (Build)	Refine and finalize the co-designed implementation strategy, ensuring feasibility, scalability, and alignment with existing systems.	Workshop with 20 young adults and 20 caregivers to refine the implementation strategy Prototyping and iterative refinement Feedback workshop with 15 government stakeholders and NGOs Finalize culturally adapted, sustainable implementation strategy	Finalized co-designed implementation strategy ready for feasibility testing for broader implementation

a PSS: psychosocial support services.

b AYA: adolescents and young adults.

c KZN: KwaZulu-Natal.

d DoE: Department of Education.

e DoH: Department of Health.

f DSD: Department of Social Development.

g NGO: nongovernment organization.

h FGD: focus group discussion.

#### Phase 1: Discover

The first phase of the study aims to establish a foundational understanding of the current mental health and PSS landscape in KZN. This phase is informed by findings from a separately conducted systematic review of evidence-based mental health interventions for adolescents and young adults in SSA. The methods and results of that review are reported in a dedicated manuscript currently under review. It assesses the availability and accessibility of existing mental health and PSS services while identifying key barriers that limit their use. A particular focus is placed on young adults’ perspectives, recognizing the importance of their engagement in shaping implementation strategies that directly impact them.

Qualitative inquiry is a key component of phase 1. The first qualitative inquiry will be an inception meeting held with government stakeholders and NGOs. This meeting will serve as a foundational engagement with relevant stakeholders from the DoE, DoH, DSD, and implementing partners (NGOs) to gather insights on the current mental health and PSS services available to adolescents and young adults and to identify challenges and barriers in implementing these services.

The second step in this phase will be workshops held with young adults and caregivers. The workshops will include journey mapping exercises and focus group discussions (FGDs) to explore their lived experiences and perceptions of mental health services, their needs, and barriers to accessing mental health services. To enhance understanding, participatory methods such as journey mapping will be used to allow young adults to visually depict their experiences with mental health challenges and care, from identifying a need for support to seeking and receiving care.

Three workshops will be conducted in this phase. The first workshop will be a pilot workshop that aims to test the designed tools (Multimedia Appendix 1).

#### Phase 2: Design

The second phase will focus on the co-design of an implementation strategy using a structured HCD approach. Co-design is conceptualized as a collaborative and participatory process that brings together individuals with lived experience of mental health challenges, service providers, and design experts to collectively develop innovative solutions [37]. This approach emphasizes shared decision-making, mutual learning, and inclusivity, ensuring that those directly affected by mental health issues have an active role in shaping interventions, policies, and services. While young adults and their caregivers with lived experience provide critical insights into real-world challenges and needs, consulting with service providers will contribute clinical expertise and practical knowledge, and design experts will facilitate the creative process, guiding the group through structured methodologies to generate meaningful, user-centered solutions. This cocreation process fosters a sense of ownership, empowerment, and responsiveness in mental health service design, ultimately leading to implementation strategies that are more effective, relevant, and sustainable. For this to happen, all stakeholders need to be constantly kept informed by sharing findings.

Findings from phase 1 will be presented to stakeholders in 2 separate workshops with young adults and caregivers. The process will center on ideation and problem definition, where young adults and their caregivers, as the end users, will be guided through a facilitated brainstorming workshop to generate implementation ideas that address mental health and PSS service challenges. Ideas will be prototyped within groups, and the most appropriate implementation strategy determined within a plenary session will be selected [38].

#### Phase 3: Build

This phase will focus on refining the implementation strategies and involve structured feedback loops, ensuring that the proposed solutions are both feasible and responsive to the needs of young adults. This will be done in 3 workshops. One feedback workshop will engage young adults separately, similarly another workshop with caregivers, and the final workshop will engage government and NGO stakeholders. The workshops with young adults and caregivers will focus on finalizing the co-designed implementation strategies following insights gained during the first and second phases. This will include prototyping through scenarios and iterative refinement, where participants will work together to finalize the implementation strategy. This implementation strategy may take various forms, such as a service delivery framework, a referral pathway peer support model, or a school-based screening tool. The workshop with stakeholders from the DoE, DoH, DSD, and the NGOs will involve presenting the co-designed implementation strategies for their insights. The stakeholders will assess the proposed solutions and prioritize the most practical and accessible components. Discussions will focus on how best to integrate these strategies within existing health and education systems, including policy alignment, training requirements, and referral pathway development. This process will help add valuable insights from the service provider experts on feasibility, acceptability, and effectiveness. By the end of this phase, the study will have produced a comprehensive, co-designed implementation strategy that is informed by stakeholder input, culturally adapted, and structured for sustainable implementation.

### Sampling Recruitment

#### Overview

Participants for this phase will be selected using a criterion purposive sampling strategy to ensure representation from multiple stakeholder groups. A criterion-based purposive sampling approach [37], drawing from an existing Asenze longitudinal cohort, involves identifying and selecting individuals or groups of individuals on the assumption that they are knowledgeable about or experienced with a phenomenon of interest. Participants will be selected using inclusion criteria comprising active cohort enrollment, being in one of two defined participant groups—adolescents and young adults or caregivers—and their availability and willingness to participate across all workshop phases while sharing mental health experiences. All decisions regarding sampling of participants will be discussed and made by the study’s research team.

This approach aligns directly with the study’s objectives. Criterion-based selection of adolescents and young adults and caregivers with lived experience of community mental health services will ensure information-rich contributions to service mapping (objective 1) and barrier identification (objective 2), while the deliberate inclusion of structurally diverse participant groups—adolescents and young adults, caregivers, and institutional stakeholders from the DoE, DoH, and DSD will introduce maximum variation in perspective, which can show barriers across individual, familial, and systemic levels simultaneously. More importantly, drawing from an existing cohort some of whose members include those with prior experience to some mental health concepts through earlier study phases will enhance the depth of co-design contributions (objective 3). Retaining the same participants across the Discover, Design, and Build phases will also ensure that feasibility and acceptability judgments (objective 4) will be grounded in the same contextual knowledge that will shape the delivery models’ design.

The study will purposively sample 20 young adults between the ages 18 and 22 years who have experienced CMHDs as adolescents (corresponding to their age ranges when participants in the Asenze study during waves 3 and 5), 20 caregivers, and 15 stakeholders (from the DoE, DoH, and DSD, along with NGO implementation partners, namely Bridging the Gap South Africa, South African Depression and Anxiety Group, the President’s Emergency Plan for AIDS Relief, the KZN Civil Society and Civil Society Youth Sector, and the KZN Office of the Premier) based on qualitative sampling guidelines that prioritize depth, diversity of perspectives, and the achievement of thematic saturation [39].

In qualitative research, data saturation, the point at which no new themes emerge, is commonly achieved with sample sizes ranging from 12 to 20 participants per homogeneous group, depending on the scope and complexity of the research question [39]. Thematic saturation will be assessed by following Braun and Clarke’s [40] iterative and reflexive process. Given that the study uses a multigroup participatory design, moving sequentially from the Discover through the Build phase, saturation will be assessed within and across groups and phases, recognizing that adolescents’, young adults’ and caregivers’ data may saturate at different points and that saturation within one phase does not necessarily imply saturation of the full analytical account. This means themes identified in earlier phases are deliberately revisited in subsequent phases, providing an iterative check on whether the analytical account is sufficiently developed before data collection concludes.

Purposive sampling within each group ensures inclusion of participants with relevant lived or professional experience, and the sample sizes were determined to ensure breadth of representation while maintaining feasibility for interactive HCD workshops. This aligns with recommendations for participatory design studies, which benefit from smaller, focused group sizes that allow for in-depth engagement and iterative feedback [41].

#### Young Adults Inclusion Criteria

To ensure we recruited eligible young adults who had experienced mental health challenges and are still part of the Asenze cohort, we identified participants from the Asenze longitudinal study who had completed both wave 3 and wave 5 screening procedures. The inclusion criteria for the young adults were:

- Male and female young adults aged >18 years.
- Were enrolled in Asenze wave 3 and screened positive for either depression, anxiety, and/or suicidal ideation through the Patient Health Questionnaire 9-item (PHQ-9) or the Generalized Anxiety Disorder 7-item (GAD-7) questionnaire during their enrollment, with cutoff scores for inclusion being PHQ-9, moderate to severe depression (total score between 10 and 20-27) and/or GAD-7, moderate to severe anxiety (total score between 10 and 15+).
- Be in the Asenze wave 5 cohort and completed the screening process using the PHQ-9 or the GAD-7 questionnaire, with cutoff scores for inclusion being PHQ-9 moderate to severe depression (total score between 10 and 20+) and/or GAD-7 moderate to severe anxiety (total score between 10 and 15+).
- Must have self-identified through initial recruitment contact to have experienced any of the mental health challenges previously or recently.
- Available to participate through all phases of the study.

We applied inclusion criteria based on the PHQ-9 and GAD-7 screening tools, as well as referrals for suicidal ideation. Anxiety and depressive symptoms were measured using the GAD-7 [42] and the PHQ-9 modified for Adolescents (PHQ-A) [43], respectively. Both instruments have demonstrated good psychometric properties and have been validated for use in South African settings, including among adolescents [44-46]. In the wave 3 Asenze sample, the GAD-7 demonstrated acceptable internal consistency (*α*=.78), and the PHQ-A demonstrated good internal consistency (*α*=.81).

**Figure 2.**
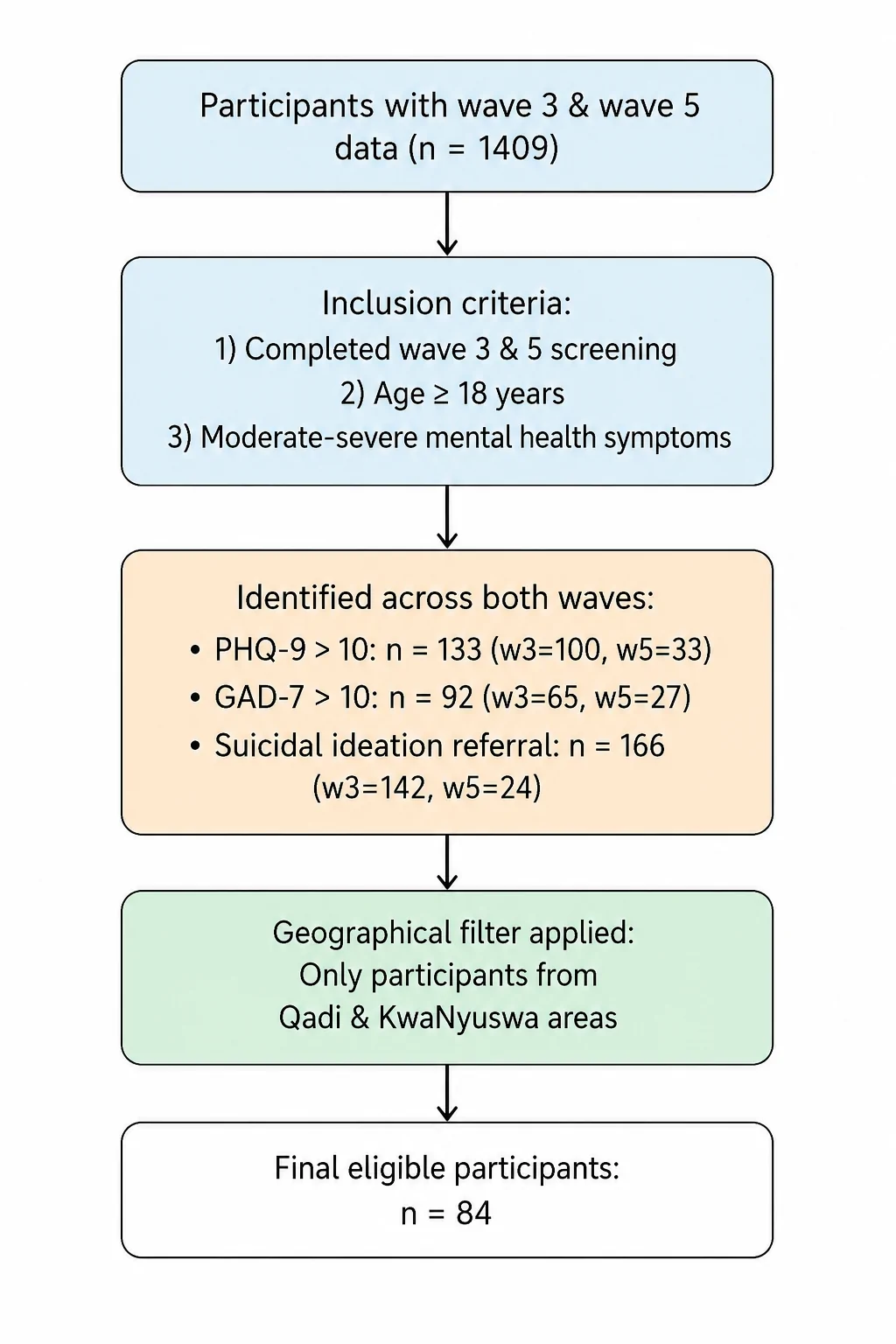
Illustration of the young adults’ recruitment flow. GAD-7: Generalized Anxiety Disorder 7-item; PHQ-9: Patient Health Questionnaire 9-item.

When incorporating data from both waves, we identified 133 participants who had a PHQ-9 score of >10, indicating moderate to severe depression (100 participants from wave 3 and 33 from wave 5). Similarly, we identified 92 participants with a GAD-7 score of >10, reflecting moderate to severe anxiety (65 from wave 3 and 27 from wave 5). Additionally, 166 participants were referred for suicidal ideation across both waves (142 from wave 3 and 24 from wave 5). However, this data represented participants from the different areas in the Valley of a Thousand Hills, which is a vast region with long traveling distances. For feasibility and accessibility, the data were filtered to include eligible participants from the Qadi and KwaNyuswa areas, which are geographically close to each other. Although geographically restricted, these communities reflect the broader perirural characteristics of the Valley of a Thousand Hills and therefore remain appropriate for co-design purposes. This then yielded 84 participants in total as indicated in Figure 2, which illustrates the recruitment process.

#### Caregiver Inclusion Criteria

For the caregivers, their inclusion criteria are that they must be providing care to the 20 recruited young adults at the time of recruitment and be available to participate in all phases of the study. They should also be willing to share any experiences they may have with their young adults’ mental health challenges and their own challenges in a group setting.

#### Stakeholder Inclusion Criteria

Government stakeholders from DoE, DoH, and DSD, as well as implementing NGO partners from provincial and local government, will also be among our study population. However, these stakeholders will not participate in all phases. There will be an inception meeting held separately in phase 1 with the stakeholders to introduce the study, share ISHP and Asenze’s main study findings on mental health, and get a sense of the available and accessible mental health and PSS services they provide for adolescents and young adults in KZN. We will engage them again in phase 2 to provide feedback from workshops in phase 1. Then we will hold a workshop in phase 3 to get their input on the co-designed implementation strategies.

#### Young Adults and Caregivers Recruitment

Young adults and caregivers will be recruited through the following procedures:

1. We will use the Asenze wave 3 and 5 data to preidentify eligible participants who have completed the assessment.
2. Contact the caregivers of the identified young adults to inform them telephonically of the Asenze Impilo study.
3. Contact the identified young adults and caregivers for recruitment to participate in the study. The researcher and research assistant will use updated contact information from the Asenze wave 5 database. Contact will involve a phone call to explain the study and its focus on discussions around experiences of mental health challenges. If interested in participating, we will schedule participation in workshops.
4. Confirm young adults’ and caregivers’ availability and suitable days.
5. We will call and send SMS reminders 48 hours before the scheduled workshop dates.

#### Stakeholder Recruitment

Stakeholder recruitment will involve the following steps:

1. Use prior contact information obtained through the ISHP evaluation project to contact relevant departments and directorates responsible for mental health and PSS services.
2. Arrange an initial online meeting with stakeholders from DSD, DoH, and DoE to introduce and inform them about the study. We will leverage these online meetings to connect with other stakeholders based on information from the initial online meetings, then invite those who are willing to be part of the co-designing process to an in-person inception meeting.
3. Send a formal request to the head of departments for permission to work with members from their departments.
4. Send out a formal invitation letter with details of the inception meeting and obtain confirmation via email.
5. Send out an email reminder a week before the scheduled date.

### Study Setting

The study is taking place in KZN, one of South Africa’s 9 provinces, situated in the southeastern part of the country. The demographic composition of KZN is predominantly Black African, with many residents speaking isiZulu as their first language [47].

Administratively, KZN is structured into 10 district municipalities: Ugu, uMgungundlovu, uThukela, uMkhanyakude, King Cetshwayo, Harry Gwala, uMzinyathi, Amajuba, Zululand, and iLembe. Additionally, eThekwini functions as a metropolitan municipality, encompassing the province’s largest urban center, Durban. Within this administrative and demographic landscape, various regions face distinct socioeconomic and health-related challenges, shaping the context in which research and implementation strategies take place [47]. One such region is the Valley of a Thousand Hills, where the Asenze study cohort is conducted, a geographically diverse area characterized by a mix of periurban and rural settlements approximately 45 km northwest of Durban.

### Data Collection and Management

The data will be collected through journey maps, card sorting exercises, and FGDs, which are recorded, and audio recording will be transcribed and analyzed thematically. All recorded data are downloaded onto a secured laptop and stored in a secure office together with all paper-based data, and access will be restricted to authorized research personnel. All data collected digitally will be stored using encrypted electronic devices and on secure servers. Identifiable information will be anonymized during the analysis phase.

The data will be collected through qualitative methods, including journey mapping, card sorting exercises, and FGDs. These sessions will be audio-recorded and transcribed. Data collection will also include visual and textual documents generated by participants during co-design activities, such as journey maps, service knowledge, and ranked service preferences from card sorting. Each transcript and artifact will be assigned a unique identifier to ensure confidentiality and facilitate data tracking during analysis.

In the Discover phase, data collection will include participatory and interactive methods designed to elicit rich insights from both young adults and caregivers on their experiences with mental health challenges and service use. Tools will include semistructured workshop guides to facilitate group discussions (Multimedia Appendix 2), journey mapping templates, and service evaluation checklists. Facilitators will be guided by a structured set of questions to ensure consistency, safety, and openness, incorporating interactive elements such as flashcards to prompt discussion about emotional and physical well-being.

In the Design phase, co-design workshops with young adults and caregivers will translate phase 1 findings into context-specific, culturally relevant, and feasible mental health and PSS implementation strategies. Facilitators will begin by recapping phase 1 results and prompting participants to articulate their “biggest worry” and “biggest hope” before exploring ideal mental health services and perceived barriers (Multimedia Appendix 3). A central activity will be the card sorting exercise, where participants will review a set of implementation strategy feature cards, ranging from school-based counseling and peer support groups to mobile clinics and digital referral tools, and rank them by importance and preference. Small group discussions will then compare choices, explore reasoning, and reach consensus on priority features. These priorities will inform structured problem-solving discussions on practical implementation, considering delivery settings, responsible actors, required resources, and strategies for addressing stigma, gender-related barriers, and the needs of youth affected by HIV. Feedback sessions will capture participants’ views on the workshop process and the refined proposed implementation strategies.

In the Build phase, the focus will be on refining, testing, and planning the implementation of the co-designed mental health implementation strategies developed in earlier phases. Workshops with young adults and caregivers will present an opportunity to present 3 prototype co-designed implementation strategies, using interactive feedback stations and scenario-based role plays to explore their relevance, safety, and feasibility (Multimedia Appendices 4 and 5). Participants will be encouraged to interact with real-life scenarios to assess each model’s potential benefits, risks, and acceptability, including stigma considerations and suitability for youth affected by HIV, gender-based violence, or other vulnerabilities. This will be followed by group discussions to identify strengths, gaps, and suggestions for improvement, followed by voting activities to prioritize key features or preferred implementation strategies. Feedback will be documented through structured templates and reflection activities, while visual aids (posters) will be used to help communicate each implementation strategy’s purpose.

To ensure that these diverse stakeholder inputs are translated into concrete implementation strategy components, each workshop phase will use structured facilitation tools, including HCD techniques such as card sorting and preference ranking, to capture and prioritize feedback, preventing dominant voices from suppressing minority perspectives. Following each workshop, the research team will debrief and discuss the feedback, mapping each proposed strategy component against dimensions of acceptability, feasibility, safety, and cultural appropriateness, as shared by the participant group. Points of convergence across adolescents and young adults and caregiver groups will be incorporated as core design features, while points of divergence will be escalated to deliberative resolution in subsequent workshops.

In phase 3, refinement workshops with adolescents and young adults and caregivers will produce specific tangible outputs, including a finalized implementation strategy, suggested participant-validated implementation protocols, and documented design constraints reflecting features explicitly rejected by either group. Government and NGO stakeholder workshop will serve a distinct function, that is, stress-testing the draft implementation strategies against policy and resource realities, producing a policy alignment matrix and feasibility gap analysis that will together inform an integration planning process. Final synthesis will triangulate outputs across all stakeholder groups, ensuring that the final implementation strategy is simultaneously grounded in community lived experience, operationally feasible, and policy-coherent.

All audio files and scanned documents will be downloaded to encrypted, password-protected laptops and a USB and securely backed up on a university-approved cloud server. Physical documents, for example, workshop notes, participant feedback templates, and participant maps, will be stored in locked cabinets in a secure office accessible only to authorized research personnel. All identifying information will be removed or anonymized at the transcription stage to enhance safety, confidentiality, trustworthiness, and transparency, as recommended for qualitative research protocols [41,48].

### Ethical Considerations and Risk Management

This study has applied for ethical approval from 2 institutional ethics bodies, the University of KwaZulu-Natal’s Biomedical Research Ethics Committee (BREC/00006912/2024) and Columbia University’s Institutional Review Board (IRB-AAAU7173). Approval from both institutional ethics boards has been granted. All participants will be provided with a copy of an informed consent form, which they can keep. To collaborate with stakeholders from the DoH, DoE, DSD, and their implementing partners, letters of approval have been sought from the Head of Department of the 3 ministries, together with supporting letters from the participating NGOs.

All young adults will be aged 18 years or older when recruited to participate in the study. Young adults and caregivers’ interest in participating will initially be assessed via telephonic contact, followed by written consent on an informed consent form at the first workshop. Consent forms will be securely stored and transported to a secure location. Additionally, to avoid linking the new study numbers to the Asenze study numbers, both study identifiers were stored and locked separately.

The anticipated risks for participants are minimal. The primary concern is the potential loss of confidentiality and the possibility of emotional distress when discussing sensitive mental health topics. Workshops will be held in private venues accessible only to consenting participants and authorized staff. At the start of each session, participants will be reminded that while confidentiality is expected, it cannot be fully guaranteed in a group setting, and they will be encouraged to avoid sharing information they would not be comfortable disclosing. They will also be reminded not to share any information shared during the workshops outside of the study.

Given that the participant pool includes young adults with prior moderate to severe mental health symptoms, a robust protocol for managing distress and adverse events will be in place and will include:

- On-site clinical support: a qualified clinical psychologist and a supervised psychology intern will be present at all workshops to provide immediate clinical expertise, crisis intervention, and emotional support as required.
- Real-time monitoring: facilitators will be trained to identify early signs of participant distress and will discreetly alert the psychologist or intern for immediate assessment.
- Immediate referral mechanisms: if a participant exhibits acute distress, suicidal ideation, or other high-risk symptoms, the on-site psychologist will conduct an immediate risk assessment and, if necessary, refer the participant to appropriate services. Referral pathways will include Lifeline National Crisis Line, the South African Depression and Anxiety Group, local clinics, and other specialized mental health services in the area. Any adverse event or serious distress incident will be documented and reported to the principal investigator.
- Private debriefing space: a quiet, private space will be available for one-on-one debriefing and support away from the main group if required.
- Follow-up support: the clinical team will follow up on participants with any adverse incidents requiring follow-up, liaising with local service providers to ensure continuity of care.

Data collection materials will be translated into isiZulu and English, and workshops will be facilitated by researchers and research assistants familiar with community and cultural norms in KZN, ensuring participants can engage in their preferred language.

### Data Analysis

Qualitative data analysis will follow an inductive and iterative thematic analysis approach [40,48], guided by the principles of HCD and participatory research. The analysis will seek to identify key patterns, needs, and barriers related to mental health and PSS services among adolescents and young adults in KZN. The qualitative data from the service mapping exercise, journey mapping, FGDs, and card sorting workshops will be analyzed thematically using the latest NVivo software (version 15; Lumivero). The analytic process will follow the 6 phases of thematic analysis as outlined by Braun and Clarke [40], allowing for an inductive and iterative exploration of patterns, experiences, and concerns related to mental health and PSS services.

Phase 1 will involve familiarization with the data, during which the research team will read and reread transcripts, review visual outputs, and note initial observations to build a deep understanding of participants’ lived experiences. In phase 2, generating initial codes, both semantic and latent codes will be developed inductively, allowing insights to emerge directly from the data [49]. NVivo will be used to systematically apply these codes across the dataset. Phase 3, searching for themes, will involve grouping related codes into broader categories that capture important patterns. This will be followed by phase 4, reviewing themes, where potential themes will be refined, ensuring they accurately represent the data and are coherent both internally and in relation to the overall dataset.

In phase 5, defining and naming themes, the research team will articulate the essence of each theme and determine how it relates to the study objectives and the broader literature on mental health service access, acceptability, and quality. Finally, in phase 6, producing the report, the themes will be synthesized into a coherent narrative supported by illustrative quotes and visual materials from journey maps and card sorting outputs. This approach aligns with the participatory and user-centered principles guiding the study and ensures that the analysis remains grounded in the perspectives of young adults, caregivers, and service providers.

Data generated during the Build phase, including feedback on implementation strategy prototypes, feasibility and acceptability assessments, and service provider evaluations, will be analyzed using the same reflexive thematic analysis framework applied in the Discover and Design phases [40]. This will ensure analytic consistency across the study. However, rather than generating stand-alone thematic reports, the Build phase analysis will focus specifically on identifying merging and deviating feedback and themes established in earlier phases. The similar findings will be incorporated as finalized implementation strategy components, while the deviating findings will undergo review. Any quantifiable preference and rating data collected through structured feedback tools will be summarized descriptively to complement qualitative findings. The integrated output of this analysis will be a finalized, evidence-informed, and community-validated implementation strategy that reflects the cumulative analytical work across all 3 phases.

## Results

The study was funded in 2024, and ethics approval was received in March 2025. The enrollment process will be completed in April 2025; then, data collection will be conducted between May 2025 and January 2026. Data analysis will follow at the end of data collection, and the first results are expected to be submitted for publication in 2026.

## Discussion

### Overview

Expected outcomes of this study include the development of a context-specific implementation strategy for mental health and PSS with the possibility of integration within either the health or education system, enhanced collaboration among stakeholders for sustainable mental health service delivery, and policy recommendations for improving young people’s mental health care in South Africa. Although the study engages a purposively selected group of 15 mental health stakeholders, their inclusion from the DoH, DoE, and DSD ensures representation of key institutional perspectives central to service delivery [50]. This focused, cross-sectoral engagement allows for in-depth exploration of coordination challenges and facilitators within existing systems, providing insights that are intended to support future scale-up in similar provincial and national contexts.

Additionally, the study aims to strengthen the capacity of local service providers to provide youth-friendly mental health services, improve early identification and implementation mechanisms, and contribute to knowledge on best practices for co-designing mental health implementation strategies. The findings will also inform practical frameworks and tools that can be adapted by nonparticipating institutions, thereby supporting broader capacity-building beyond the immediate study sites.

### Strengths and Limitations of the Study

The study has several strengths. By using an HCD approach, it ensures that implementation strategies are cocreated with direct input from young adults, caregivers, and government and their implementing partners, making them contextually relevant and user-friendly. The participatory, mixed methods design allows for a comprehensive understanding of mental health service gaps, leveraging both qualitative insights and quantitative data. Additionally, the study builds upon the robust longitudinal data from the Asenze study, providing a strong evidence base for implementation strategy development. Stakeholder collaboration with government departments and community organizations further strengthens the study’s potential for policy integration and long-term impact.

However, the study also has some limitations. The findings may be context-specific and may not be easily generalizable beyond KZN due to regional differences in service availability and sociocultural factors. In addition, the study relies on self-reported data from young adults and caregivers, which may be subject to recall bias or social desirability bias. The HCD process can be time-consuming, especially in this study, where the process was implemented by a research team who were both learning and executing the process as the project unfolded. Despite these limitations, the study’s participatory approach and stakeholder engagement strategies will enhance its potential for sustainable impact.

### Conclusion

This protocol outlines a rigorous, participatory process for developing an implementation strategy for delivering evidence-based mental health and PSS tailored to the needs of adolescents and young adults in perirural KZN, South Africa. By combining the SEM and HCD, the study ensures that solutions address individual, interpersonal, community, and policy-level determinants of mental health while remaining grounded in lived experience. Through collaboration with young adults, caregivers, and government and NGO stakeholders throughout all phases, the study is expected to produce an implementation strategy that is culturally relevant, feasible within existing systems, and responsive to local priorities. Ultimately, the implementation strategy can be evaluated for effectiveness. Efforts like these are needed to strengthen mental health services in low-resource settings and support the well-being of South African adolescents and young adults.

## Supplementary material

10.2196/90096 Multimedia Appendix 1 A guide with possible questions to be used for the pilot workshop with the adolescents and young adults.

10.2196/90096 Multimedia Appendix 2 A guide with possible questions to be used in the Discover workshop with adolescents and young adults and caregivers.

10.2196/90096 Multimedia Appendix 3 A guide with possible questions to be used in the Design workshop with adolescents and young adults and caregivers.

10.2196/90096 Multimedia Appendix 4 A guide with possible questions to be used in the Build workshop with adolescents and young adults and caregivers.

10.2196/90096 Multimedia Appendix 5 A guide with possible questions to facilitate discussions with mental health stakeholders as part of the Build phase.

## Abbreviations

CMHD: common mental health disorder
DoE: Department of Education
DoH: Department of Health
DSD: Department of Social Development
FGD: focus group discussion
GAD-7: Generalized Anxiety Disorder 7-item
HCD: human-centered design
ISHP: Integrated School Health Policy
KZN: KwaZulu-Natal
LMIC: low- and middle-income country
NGO: nongovernment organization
PHQ-9: Patient Health Questionnaire 9-item
PHQ-A: Patient Health Questionnaire 9-item modified for adolescents
PSS: psychosocial support
SEM: social ecological model
SIAS: Screening, Identification, Assessment, and Support
SSA: sub-Saharan Africa
