# Supplementary material for: Enhancing Mental Health and Psychosocial Support for Adolescents and Young Adults Through the Asenze Impilo Project: Protocol for a Human-Centered Design Study
Source: JMIR Res Protoc. 2026 Sep 10;15:e90096. doi: 10.2196/90096 (PMC13561444; doi:10.2196/90096)
Supplement: Supplementary file 1 [file resprot-v15-e90096-s001.docx]

Appendix A: Discover Workshop 1- AYAs Guide (Pilot)

**Participant Information**

- **Participant Asenze ID/NAME:** _____________________
- **Gender:** ☐ Male ☐ Female
- **Facilitator Name:** ___________________
- **Date:** ___________________

**SECTION 1: Understanding Life Challenges & Mental Health Impact (30Min)**

1.1 Personal Experience

Can you tell me about some of the biggest challenges or pressures you’ve faced recently?

What does a stressful day or period in your life usually look like?

1.2 Emotional Impact

When things get tough, how does it usually affect you emotionally or mentally?

Have there been times when things felt overwhelming or hard to manage mentally? What was going on at the time?

1.3 Coping Mechanisms

When you’re feeling low or stressed, what do you usually do to cope or feel better?

Who or what do you turn to when you’re struggling?

**SECTION 2: Exploring Attitudes Toward Mental Health Support (30min)**

3.1 Personal Views

What does ‘mental health’ mean to you?

Have you or someone you know ever thought about speaking to someone (like a counsellor or psychologist)? What made it easier or harder to do that?

3.2 Social and Cultural Influences

How do people around you – family, friends, community – usually respond when someone talks about struggling mentally or emotionally?

Are there any messages you’ve received (from school, media, religion, etc.) about mental health or asking for help?

**LUNCH (30Min)**

**SECTION 3: Journey Mapping Exercise (1Hour)**

**Instructions:**

- Reflect on your experiences or imagine what it might be like for someone your age seeking help when struggling with mental health, for example, “feeling down”, “feeling low and no energy”, or “struggling” emotionally.
- Use the prompts below to map out the steps, emotions, and challenges involved in the journey.
- You can write or draw your journey in the provided large sheets.
- Follow the timeline below to reflect on your experiences.

**Stage 1: Recognising the Issue/Reason for seeking help**

Think about your mental health journey or your interactions with PSS services. Draw a step-by-step map of your experiences (e.g., moments when you sought help, interacted with a counsellor, nurse, teacher or faced challenges).

- What happened on the day you felt like you needed help? How long have you been feeling this way? When did it start? Have you felt like that before?
  *(Write or draw)*

_____________________________________________________________________

- How did you feel? (Draw or show in any preferred way on your map)
  ☐ Confused ☐ Scared ☐ Sad ☐ Angry ☐ Ashamed ☐ Glad ☐ Relieved ☐ Other:________________________________________________________________
- Who (if anyone) did you talk to first? Were there more than one person?
- How did you feel after this initial talk?

_____________________________________________________________________

**Stage 2: Seeking Help**

Mark the moments or steps that were most helpful or positive. Write or draw what made them so. Highlight the most difficult or challenging moments in this process (use different colours). What made them challenging? Show these on your map. Use colours, symbols, or words to express your emotions.

- What did you do to get help?

_____________________________________________________________________

- Did anything make it hard for you to get help? If yes, what made it hard? How did that make you feel?
- Did you give up, or try another way to get help?

_____________________________________________________________________

- Did anything make it easy to get help? If yes, what made it easy? How did that make you feel?

_____________________________________________________________________

**Stage 3: Accessing Services**

- Did you visit any places for help (e.g., school counsellor, peer support/learner support, clinic…)? If yes, which places? Was it Private or Public? Was it easy to go there? What happened there? Who did you talk to?

_____________________________________________________________________

- Did you get the help you were seeking? If yes, what help did you receive?
  If not, did you get a referral? Did they suggest an alternative?

_____________________________________________________________________

- How did the service(s) make you feel?
  ☐ Better ☐ The same ☐ Worse
  Why? Please explain

_____________________________________________________________________

_____________________________________________________________________

•	Do you still use this service/support when you are struggling? Why?

If not, why? What support are you getting now/service are you using now?

_____________________________________________________________________

**Stage 4: Follow-Up Support**

- Did anyone check in with you after you got help? If yes, who? What support did they offer?

_____________________________________________________________________

- How did that make you feel?

_____________________________________________________________________

- What support do you wish you had received as a follow-up? (Use specific their examples from the mental health exercise)

_____________________________________________________________________

**BREAK (10Min)**

**Section 4: Group Presentations (1h30min)**

**Reflection Questions**

**Note: Ensure that the audio recorder is positioned well for presentations**

1. What would you change about your experience?
2. What advice would you give to someone else going through something similar?

**Section 5: Implemetation Strategy Awareness Checklist and Feedback (30Min)**

**Instructions:**

Check whether you’ve heard about or used the services below. For services you’ve used, tell us why.

| **Service** | **Heard About It?** | **Used It?** | **Purpose/Reason for Use** |
| --- | --- | --- | --- |
| School Counselor | ☐ Yes ☐ No | ☐ Yes ☐ No | ______________________________________________________________ |
| Peer Support Groups | ☐ Yes ☐ No | ☐ Yes ☐ No | ______________________________________________________________ |
| Mental Health Helpline | ☐ Yes ☐ No | ☐ Yes ☐ No | ______________________________________________________________ |
| Community Clinic | ☐ Yes ☐ No | ☐ Yes ☐ No | ______________________________________________________________ |
| Religious/Spiritual Help | ☐ Yes ☐ No | ☐ Yes ☐ No | ______________________________________________________________ |
| Other: |  |  |  |

**Feedback**

How did you find the workshop?

Were the questions easy to follow and understand?

Was the duration of the workshop fine with you?

What can we do to improve the workshop?

What can we do to get you to engage/participate more?
