# Supplementary material for: Enhancing Mental Health and Psychosocial Support for Adolescents and Young Adults Through the Asenze Impilo Project: Protocol for a Human-Centered Design Study
Source: JMIR Res Protoc. 2026 Sep 10;15:e90096. doi: 10.2196/90096 (PMC13561444; doi:10.2196/90096)
Supplement: Supplementary file 2 [file resprot-v15-e90096-s002.docx]

Appendix B: Discover Workshop 1 – AYAs Guide

**Participant NAME: _____________________**

**Gender: ☐ Male ☐ Female**

**Facilitator Name: ___________________**

**Date: ___________________**

**SECTION 1: Understanding Life Challenges & Mental Health Impact (30 minutes)**

1.1 What does mental health mean to you?

1.2 What are the views about mental health in your community? How do people around you -family, friends, community - view someone who reaches out for help?

**SECTION 2: Revised Journey Mapping Exercise (1 hour)**

Instructions (with emphasis on help-seeking and awareness):

Picture or recall your own experience of seeking help for mental health issues or struggles, or imagine a scenario where you or a friend might need help, and draw or write your “journey” from start to hopefully healing.

Think about a time when you felt mentally or emotionally overwhelmed and needed help (recall your own experience when you faced a difficult situation). What happened? Where did you go for support? What were you feeling at each point? What helped or didn’t help?

Consider from your experience:

Did you know where to go for help (counsellor, community health centre, peer group)?/

Where would you go for help?

Did you talk to someone (teacher, parent, friend)?/

Who would you talk to?

Did you face barriers in accessing help (stigma, financial worries, not trusting services)?/

What are the barriers/challenges that would affect you getting help?

Did you find support or not?

What kind of support would you have wanted to get?

**LUNCH (30 minutes)**

**Section 3: Group Presentations (1 hour 30 minutes)**

Reflection Questions (after each presentation):

What stood out in your journey?

What was most challenging?

What made a difference (positively or negatively)?

What would you change about your experience/the scenario?

What advice would you give someone else going through something similar?

**Section 4: Implementation Strategy Awareness (30 minutes)**

4.1 Which of these services have you heard about or used?

School counsellor

Peer support groups (which ones)

Mental health helpline (which ones)

Community clinic

Religious or spiritual help (give examples)

Other (please describe)

4.2 If you used any of these services, tell us:

What was helpful?

What was not helpful?

What could have worked better?

4.3 What services have you wanted to use and could not- what were the reasons you couldn’t

4.4 If you could change one thing in the system, what would it be, and why?

**Section 5: Feedback**

How did you find the workshop?

Was the duration appropriate?

Were the questions easy to follow?

Which questions did you find challenging?

Was it helpful for you to talk openly?

In what ways might this have been helpful for you?

What can we do to make this workshop even better?

Discover Workshop 1- Caregivers Guide

**Participant Information**

- **Participant ID:** _____________________
- **Facilitator Name:** ___________________
- **Date:** ___________________

**Aim**- We want to learn more about the experiences caregivers go through when supporting an AYA’s mental health. You can reflect on your journey with your AYA or think about what a caregiver might generally experience.

If you feel comfortable, we’ll discuss these experiences as a group. You can share ideas about a general or fictional journey instead of personal details if you prefer.

**Part 1: Recognising the Problem**

- What signs made you realise your AYA might need help? How long had these signs been present?

___________________________________________________________________________

___________________________________________________________________________

- What emotions did you experience during this time? Explain.
  ☐ Confused ☐ Overwhelmed ☐ Worried ☐ Hopeful ☐ Other: ___________

___________________________________________________________________________

- Who (if anyone) did you talk to first? What was the response?

___________________________________________________________________________

- What challenges did you face during this time?

___________________________________________________________________________

**___________________________________________________________________________**

**Part 2: Seeking Help**

- What steps did you take to get help for your AYA?

___________________________________________________________________________

___________________________________________________________________________

___________________________________________________________________________

- What barriers/challenges did you face? Explain.
  ☐ Financial constraints ☐ Stigma ☐ Lack of information ☐ Other: __________

___________________________________________________________________________

- What support (if any) did you get that made it easier? From who/where?

___________________________________________________________________________

___________________________________________________________________________

**Part 3: Accessing Services**

- Did your AYA receive any professional support or services?
  ☐ Yes ☐ No

If yes:

- What type of service(s) were accessed? Was it Private or Public service?
  ☐ School counselor ☐ Clinic ☐ Community group ☐ Other: ____________

___________________________________________________________________________

- Were these services helpful?
  ☐ Yes ☐ No
  Why and how?

___________________________________________________________________________

___________________________________________________________________________

**Part 4: Supporting Your Child**

- What role did you play in supporting your AYA through their journey?

___________________________________________________________________________

___________________________________________________________________________

- What challenges did you face as a caregiver during this time? Explain.
  ☐ Balancing time ☐ Emotional strain ☐ Lack of resources ☐ Other: _____________________________________________________________________
- What kind of support did you need but did not receive?

___________________________________________________________________________

- What would you have done differently?

___________________________________________________________________________

**(OR use a fictional Journey Mapping guide)**

Let’s imagine a caregiver, Noma, who notices her teenage son, Themba, becoming withdrawn and irritable.

What might Noma notice about Themba that makes her concerned?

___________________________________________________________________________

What steps might she take to seek help for Themba?

___________________________________________________________________________

What challenges might she face as she seeks help from the health system?

___________________________________________________________________________

How might she feel during the process of seeking help for her son?

What support would she need to help her son feel better?

___________________________________________________________________________

What do caregivers like you find most challenging when supporting an AYA’s mental health?

How can services and support systems better meet the needs of your AYAs?

If you could give advice to another caregiver, what would it be?

**Service Evaluation Matrix**

**Instructions:**

List the mental health services you have sought for your AYA or that your AYA has sought for themselves. Share the reason for using these services and how helpful they were.

| **Service** | **Used?** | **Effectiveness (1 = Poor, 5 = Excellent)** | **Reason for using service?** |
| --- | --- | --- | --- |
| School Counselor | ☐ Yes ☐ No | 1 ☐ 2 ☐ 3 ☐ 4 ☐ 5 ☐ | _________________________ _________________________ |
| Peer Support Groups | ☐ Yes ☐ No | 1 ☐ 2 ☐ 3 ☐ 4 ☐ 5 ☐ | __________________________________________________ |
| Community Clinic | ☐ Yes ☐ No | 1 ☐ 2 ☐ 3 ☐ 4 ☐ 5 ☐ | __________________________________________________ |
| Religious Organizations | ☐ Yes ☐ No | 1 ☐ 2 ☐ 3 ☐ 4 ☐ 5 ☐ | __________________________________________________ |
| Helpline Services | ☐ Yes ☐ No | 1 ☐ 2 ☐ 3 ☐ 4 ☐ 5 ☐ | __________________________________________________ |
| Other: |  |  |  |

___________________________________________________________________________

**Section 5: Feedback**

How did you find the workshop?

Was the duration appropriate?

Were the questions easy to follow?

Which questions did you find challenging?

Was it helpful for you to talk openly?

In what ways might this have been helpful for you?

What can we do to make this workshop even better?
