# Supplementary material for: Enhancing Mental Health and Psychosocial Support for Adolescents and Young Adults Through the Asenze Impilo Project: Protocol for a Human-Centered Design Study
Source: JMIR Res Protoc. 2026 Sep 10;15:e90096. doi: 10.2196/90096 (PMC13561444; doi:10.2196/90096)
Supplement: Supplementary file 3 [file resprot-v15-e90096-s003.docx]

Appendix C: Design Workshop 2 – AYAs and Caregivers Guide

- **Participant ID:** _____________________
- **Facilitator Name:** ___________________
- **Date:** ___________________

Objectives:

- To identify needs and priorities for mental health and PSS implementation strategies for AYA based on Phase 1 findings.
- To co-design context-specific, culturally appropriate, and feasible implementation ideas based on AYA lived experiences and insights.

**1. Group Discussion - Barriers and Solutions:**

What does your ideal mental health service look like?

___________________________________________________________________________

___________________________________________________________________________

___________________________________________________________________________

What are the key barriers identified to accessing mental health services?

___________________________________________________________________________

___________________________________________________________________________

___________________________________________________________________________

___________________________________________________________________________

What solutions do you propose?

___________________________________________________________________________

___________________________________________________________________________

___________________________________________________________________________

___________________________________________________________________________

___________________________________________________________________________

Who should implement these solutions? And Where?

**2. Card Sorting Activity**

List the top three implementation strategies features identified:

Feature 1: ___________________________________________________________

Feature 2: ___________________________________________________________

Feature 3: ___________________________________________________________

Common themes:

___________________________________________________________________________

___________________________________________________________________________

___________________________________________________________________________

Diverging opinions:

Did any ideas feel unrealistic or hard to accept? Why?

___________________________________________________________________________

___________________________________________________________________________

___________________________________________________________________________

**3. Participant Feedback**

What did you find most useful about the session?

What was most challenging?

Was anything difficult to understand or did you feel uncomfortable at any point? Why?

What would have worked better?

What will you tell your family or friends about today’s workshop

Anything other suggestions or issues?
