# Supplementary material for: Enhancing Mental Health and Psychosocial Support for Adolescents and Young Adults Through the Asenze Impilo Project: Protocol for a Human-Centered Design Study
Source: JMIR Res Protoc. 2026 Sep 10;15:e90096. doi: 10.2196/90096 (PMC13561444; doi:10.2196/90096)
Supplement: Supplementary file 4 [file resprot-v15-e90096-s004.docx]

Appendix D: Build Workshop 3: AYAs and Caregivers Guide

**Participant ID: _____________________**

**Facilitator Name: ___________________**

**Date: _____________________________**

Objectives:

- To share and discuss the proposed mental health implementation strategies.
- To gather your views on whether the proposed implementation strategies are helpful, realistic, and relevant.
- To hear your suggestions for making the implementation strategies better and shape it into a more innovative, effective solution.

**Section 1: Proposed Implementation strategies’ Feedback Stations**

We have designed three possible implementation strategies based on your feedback and what you think would help with young people’s mental health challenges. In this section, we want to hear your thoughts/views about these implementation strategies. Walk around the room, visit each station, read about each idea and answer the following questions.

What do you think of these 3 ideas?

1. Gendered Safe Circles (bi-weekly group support sessions):

- What do you like or not like about this idea?
- Would you attend? Why or why not? (youth)
- Is this something youth in your community would use?
- What else could be added?
- Where would you want them to be located?

2. Peer Champions (trained young people who support others):

- What do you like or not like about this idea?
- Would you trust them to keep things you share with them private? (youth)
- What would make you feel safe talking to them? (youth)
- What kind of training should they receive?

3. “Speak Out Box” (Classroom Mental Health drop-in)

- What do you like or not like about this idea?
- Would you use it? Why or why not? (youth)
- Is this something adolescents and youth would use in schools? Why? Or why not?
- Would you feel safe to use such a box? What would make you feel safe or unsafe using this box? (youth)
- If you didn’t want to write your name or be identified, would you still use it? (youth)

**Section 2: Making it Better – Group Discussion**

Now that you have had a chance to look at the implementation strategies, discuss in small groups what you think are the strengths, gaps/concerns, and any suggestions for improvements. This feedback will help us refine the interventions to better suit your needs.

| **Implementation Strategy** | **Strengths Identified** | **Gaps/Concerns Identified** |
| --- | --- | --- |
| Gendered Safe Circles | 1: _________________________  2: _________________________  3: _________________________ | 1: _____________________  2: _____________________  3: _____________________ |
| Peer Champions Training | 1: _________________________  2: _________________________  3: _________________________ | 1: _____________________  2: _____________________  3: _____________________ |
| “Speak out” Box | 1: _________________________  2: _________________________  3: _________________________ | 1: _____________________  2: _____________________  3: _____________________ |

Suggestions for improvement:

What would you like to change, add or remove from these ideas?

What could make these ideas easier or safer for youth in your community?

Could any of these ideas cause embarrassment or make people afraid of being labelled ‘crazy’ or ‘sick’ (stigma)? How can we prevent that?

Any other issues/suggestions?

**Section 3: Implementation Planning – Group Discussion**

In this section, let us talk about what it would take to make these ideas work in real life. Think practically about how and where the implementation strategy could be tested, who should be involved, and what resources are needed. This feedback will be given to the implementing partners.

3.1 Where do you think the implementation strategy should be implemented? Where is a good place to test these?

3.2 Who do you think should be responsible for guiding the implementation? Who should be involved in helping to run these ideas?

3.3 What skills, knowledge, or resources do you think are needed for the implementation strategy to be successful?

3.4 What goals or milestones do you think are helpful for a successful implementation?

3.5 How will we know if it’s working? What changes would you see in your school/community if this is working well?

**Section 4: Workshop Feedback**

See the provided feedback sheet for individual feedback (a separate feedback sheet is provided).
