# Supplementary material for: Enhancing Mental Health and Psychosocial Support for Adolescents and Young Adults Through the Asenze Impilo Project: Protocol for a Human-Centered Design Study
Source: JMIR Res Protoc. 2026 Sep 10;15:e90096. doi: 10.2196/90096 (PMC13561444; doi:10.2196/90096)
Supplement: Supplementary file 5 [file resprot-v15-e90096-s005.docx]

Appendix E: Build Workshop 3 – Stakeholders Guide

**•	Facilitator Name: ___________________**

**•	Participant ID:**

**•	Date: ___________________**

**Objectives:**

- Present comprehensive findings from the three-phase HCD process, including key insights from young adults and caregivers about their mental health needs and preferred support mechanisms.
- Obtain feedback and insights on the co-designed implementation strategies (Peer Champions, Gendered Safe Spaces, and Speak-out Boxes) with stakeholders.
- Reach consensus on an optimal model based on stakeholder feedback.
- Identify strategic partnerships and actionable next steps. Review and refine the delivery models based on feedback and suggestions from participants.

**Section 1: Input on Implementation strategy 30min**

Participants to be divided into groups and discuss the following on the identified priority delivery model (Peer Champions).

Identify Strengths

___________________________________________________________________________

Potential weaknesses

___________________________________________________________________________

Potential Solutions

___________________________________________________________________________

Discussion questions:

•	How does this model fill the gaps identified?

•	How could the existing programmes (ISHP, SNES, DSD social work programmes, NGO programs/services etc) integrate this model to ensure feasibility and sustainability?:

o	What is immediately feasible within current systems?

o	What requires adaptation?

o	What is aspirational (longer-term)?

**Section 2: Risk Assessment 30min**

Rate the likelihood of a risk listed below and its impact from High (H), Medium (M), to Low (L), then discuss how to prevent and respond.

| **Risk** | **Likelihood (H/M/L)** | **Impact (H/M/L)** | **Responsibility (who?)** | **Contingency Plan** |
| --- | --- | --- | --- | --- |
| Peer Champions not recruited |  |  |  |  |
| Training and continued supervision |  |  |  |  |
| Youth don’t use services (stigma) |  |  |  |  |
| Peer  turnover |  |  |  |  |
| Funding  dries up |  |  |  |  |
| Lack of support from Community |  |  |  |  |
| Safeguarding incident |  |  |  |  |
| Inter-departmental conflict |  |  |  |  |
| Other: |  |  |  |  |
|  |  |  |  |  |
|  |  |  |  |  |

**Section 3: Input on designed model 15min**

Capacity and Support

- How do we identify potential peer champions? (deployment)
- What skills, training, personnel, or resources would be needed to implement this model effectively?
- What partnerships could strengthen effective implementation of the selected model?

Barriers and Risks

- What existing departmental resource (e.g., School Health Teams, Learner Support Agents, or Community Health Workers) could this model leverage to minimize new costs, improve accessibility, usability and sustainability?
- What would be main barriers to implementing this model (e.g., training, policy, infrastructure, coordination)?
- Could these barriers be addressed or mitigated? If yes, how?

**Lunch Break**

**Section 4: Plenary Session – Feedback from group discussions 1hr**

Each group has 10min to share feedback. After a facilitated synthesis of key recommendations emerging from the group discussions, a moment for final reflection:

- What is one thing we have overlooked?
- What would make this model stronger?
- What would make this model more realistic?
- What about alignment with existing Provincial Priorities? Integration opportunities”

**Section 5: Next steps 30min**

Commitment and next steps

- What practical steps and level of engagement could your department take to help us move this forward?
- What level of commitment (resources, coordination, personnel) can be pledged today?

‘Commitment Menu’ options: Please tick what is applicable to your department or organisation.

[ ] Technical oversight/guidance.

[ ] Training manuals and trainers

[ ] Providing physical space (e.g., schools/clinics/community hall).

[ ] Policy alignment/Standard Operating Procedure (SOP) development.

[ ] Co-branding or official endorsement.

[ ] Formalizing a pilot evaluation site.

[ ] Sponsoring monetary incentives (e.g. data/transport money)

[ ] Other (please specify):

**Section 6: Workshop Feedback**

What worked well in this session? What didn’t?

___________________________________________________________________________

___________________________________________________________________________

In what ways might the workshops have been helpful for you?

___________________________________________________________________________

___________________________________________________________________________

What could have been done differently? What worked well in this session?

___________________________________________________________________________

___________________________________________________________________________

Suggestions for future workshops:

“What Are We Missing?”:

**Final Reflection Round:**

- What is one thing we have overlooked?
- What would make this model stronger?
- What would make this model more realistic?
